# Estimating the collapse of Afghanistan’s economy using nightlights data

**DOI:** 10.1371/journal.pone.0315337

**Published:** 2024-12-13

**Authors:** Till Raphael Saenger, Ethan B. Kapstein, Ronnie Sircar

**Affiliations:** 1 Operations Research and Financial Engineering, Princeton University, Princeton, New Jersey, United States of America; 2 Empirical Studies of Conflict Project (ESOC), School of Public and International Affairs, Princeton University, Princeton, New Jersey, United States of America; Chinese Culture University, TAIWAN

## Abstract

The Taliban’s takeover of Afghanistan in August 2021 is associated with a rapid collapse of the Afghan economy. However, assessing the scale of this collapse is proving difficult as official data are scarce. To complement qualitative measures obtained through rapid surveys of the population, we employ monthly nightlights data as a proxy measure for changes in economic activity. By combining a synthetic control approach with nightlights data from neighboring countries, our analysis reveals a significant shift in Afghanistan’s economic trajectory: from positive growth to a deep recession, even considering the impact of the Covid pandemic. Our estimations suggest that Afghanistan’s GDP has declined by approximately 16% from 2020 to 2022, notably less than the World Bank’s current survey-based measure of a 28% decline in 2021 alone. In contrast to other available estimates, our reporting includes confidence intervals to convey the uncertainties surrounding these point estimates. This study showcases the potential applicability of our methodology and the use of appropriately processed monthly nightlights data in scenarios where administrative data is limited or unreliable.

## Introduction

The fall of the Government of the Islamic Republic of Afghanistan (GIROA) in August 2021 and the subsequent takeover by the Taliban has been associated with a rapid deterioration in the country’s economic conditions. Foreign journalists based in the nation’s capital Kabul reported on an economy on the verge of collapse in the days and weeks following the regime change [1]. The World Bank initially estimated that Afghanistan’s GDP had fallen by about one-third between 2020 and the end of 2021 [2], a number revised downward more recently to approximately 28% [3]. For its part, the United Nations Development Program (UNDP) estimated that the GDP decline was around 20% within a year of the Taliban takeover in August 2021 [4] and that it may reach 30% in the coming years [5]. This far exceeds preliminary estimates of the economic contraction associated with the Covid epidemic in Afghanistan, which was projected at the time to be on the order of 5 to 7% of GDP [6].

Measurements of the current size of the Afghan economy are based largely on rapid surveys with a relatively small number of respondents. For example, the World Bank’s Afghanistan Private Sector Rapid Survey was administered to 100 formal businesses over the phone and online between October 15 and November 15, 2021 [7]. It is unclear to what extent such estimates are reliable or representative in the current political and social environment. According to the World Bank, “official GDP statistics are not being produced” by the Taliban government, further complicating the task of economic analysis under conditions of data scarcity [8, p. 186].

The purpose of this paper is to complement existing measurements of the Afghan economy by using changes in nighttime lights (NTL, or nightlights) to estimate the decline in GDP, and to advance the utilization of the monthly cloud-free DNB composite VIIRS nightlights data set in the study of regional economic activities, particularly in settings where administrative reporting is scarce or non-existent. Nightlights have been widely used as proxy measures of economic activity since the innovative work of [9] and the related discussion by [10]. Recent publications have made use of nightlights to study not only advanced economies like China [11, 12] and the United States [13], but also the effects of conflict in countries such as Yemen [14] and the reliability of GDP estimates published by dictatorships [15]. Further, nightlights are utilized in other data scarce settings such as the study of the spatial distribution of economic activity in North Korea following trade sanctions [16]. We note, however, that the vast majority of existing studies compare annual nightlights measurements with GDP data and are able to make use of official statistics for bench-marking purposes, which is not possible for Afghanistan after 2020.

The novelty of our study is to use monthly NTL as the basis for a nowcast of the current Afghan GDP and to combine this with a relatively new synthetic control methodology for constructing counterfactual estimates. As part of this, we introduce a workflow to prepare and process the monthly cloud-free DNB composite VIIRS nightlights data set for using it as a proxy indicator for economic activity. Specifically, we use a weighted average of the nightlight radiance in provinces of neighboring countries such as Pakistan and Iran as the control. It is this gap between the current reality on the ground as reflected in the NTL data and the counterfactual of what might have been had the Taliban not emerged victorious that captures the entirety of the macroeconomic change since August 2021.

Our point estimate of the change from 2020 to 2022 is a 16% decline, which is notably less than the World Bank’s current estimate of a 28% decrease in 2021 alone. Unlike the World Bank and other international organizations, we further report confidence intervals around our economic measurements, highlighting the uncertainty surrounding these reported point estimates. On the importance of reporting confidence intervals, see [17]. Overall, we believe our methodology has broad implications for researchers studying economies where data are sparse, unavailable, or unreliable. In particular, our approach expands on previous works combining nightlights and synthetic controls [18] and permits researchers to obtain faster, and potentially more accurate, economic assessments than surveys, providing yet another, complementary method for economic analysis.

This paper proceeds in five sections. Following this introduction, the next section provides some more context and describes our data followed by a section which introduces our methodology. We then discuss the results of this estimation before concluding and discussing potential future research.

## Data

Prior to August 2021, Afghanistan depended heavily upon foreign aid and military spending to fuel its economy. Largely due to these inflows, which provided 45% of GDP and 75% of the government budget, the country “grew by more than 7% per year on average over 2001–2020, with GDP increasing by 180% between 2001 and 2020 [while] real per capita income increased by 75% between 2002 and 2018” [2, p. 1]. Foreign assistance provided direct budget support to the government, enabling the provision of services such as health care and education. Military spending boosted consumption and reduced poverty, especially where military activity was most intense [19].

Even before the Taliban takeover, the Afghan economy went into lockdown during the Covid epidemic in 2020. Yet owing to the rural character of the Afghan economy, the effects were perhaps less acute than those felt in more industrialized societies. As previously noted, the World Bank at the time projected a fall in GDP of somewhere between 5 and 7% [6]. This decline is not reflected in the currently available GDP data for 2020 [3]. Following the takeover, however, the Afghan economy faced multiple shocks, including the loss of external support, the loss of access to Central Bank foreign currency holdings due to Western sanctions, the flight of human capital, and a likely reduction in domestic private sector investment due to pervasive uncertainty about the nation’s future. Absent reliable data, measuring the scale of these shocks poses a challenge to analysts.

To overcome this data paucity, we utilize nightlights in the form of satellite-derived radiance to estimate economic output. Specifically, we use Visible Infrared Imaging Radiometer Suite Day-Night Band (VIIRS DNB) data [20]. This generation of nightlight sensors was originally launched in 2011 and presents a substantial improvement in measurement accuracy, range, and spatial resolution over the earlier Defense Meteorological Satellite Program (DMSP) [21, 22]. The measurements are available as GeoTiff (geo-referenced Tiff) files based on images of the earth’s surface that are captured in a 3000 km swath at a resolution of 500 meters at nadir. The surface of the earth is effectively divided into a grid and the observed radiance of these grid fields are reported as matrices of raster data in nanowatts per steradian per square centimeter (nW ⋅ sr^−1^ ⋅ cm^−2^).

While there are many GeoTiff data sets available, we utilize monthly stray-light corrected cloud-free composites from January 2015 to December 2022. Additionally, we use annual VIIRS nightlights (VNL) 2.1 average-masked data from 2014 to 2021 which employ an adaptive, multi-year data range threshold to remove extraneous features such as biomass burning, auroras, and background noise [23]. The remaining lights captured in these composites are primarily associated with urban economic activity [21]. We have not found any evidence of Taliban-imposed curfews or restrictions on economic activity that might disproportionately affect the nightlights measured post Taliban takeover. Afghanistan imports approximately 80% of its electricity. Despite ongoing payment difficulties, the country’s key suppliers, Uzbekistan and Tajikistan, did not halt their exports and only briefly reduced them during the time under consideration [24, 25]. Given this relatively stable supply of electricity, we expect nightlights to provide an insightful proxy to economic activity in Afghanistan during this tumultuous time period, even with reports of occasional power outages and cuts. Lastly, note that the appropriate monthly and annual observations were also available for 2014 and 2013 respectively, yet we chose to start our analysis at 2015 as we found this to yield more accurate fits of our pre-treatment synthetic control as described in the following methodology and results section. We attribute this to Afghanistan-specific shocks in 2014 such as the transition of power from the NATO-led International Security Assistance Force (ISAF) to the Afghan National Security Forces (ANSF) [26].

Beyond nightlights data, we also use the available annual GDP data from 2015 to 2022 for Afghanistan, as well as Armenia, Azerbaijan, Bangladesh, Bhutan, Georgia, India, Iran, Iraq, Jordan, Kazakhstan, Kyrgyzstan, Lebanon, Sri Lanka, Nepal, Pakistan, Syria, Tajikistan, Turkmenistan, Turkey, and Uzbekistan, which we retrieved from the World Bank online database. At the time of writing, annual GDP data were not available for Bhutan, Lebanon, and Syria in 2022. We also exclude the World Bank’s preliminary, survey-based estimate for Afghanistan’s GDP in 2021. Lastly, note that there was an outage of the VIIRS sensors collecting the data for the monthly DNB composite data in August 2022 and the available composite measurement for that month was generated based on alternative data sources. This appears to coincide with slight drop in brightness throughout the region under consideration, but since it only affects a single month, we assume the impact of this on our findings is negligible.

### Data processing and extraction

**Fig 1:**
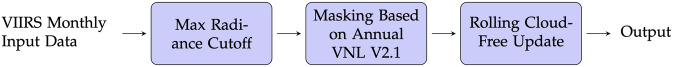
Processing flow of the monthly cloud-free DNB composite VIIRS nightlights data.

Overall, we process the monthly Monthly Cloud-free DNB Composite VIIRS nightlights data in a three-step process to prepare it for our analysis as shown in Fig 1. First, we utilize the annual VNL 2.1 data to reduce the noise of the monthly cloud-free composites as follows. The annual data has been pre-processed to identify and filter out nightlights that are most likely not associated with economic activity including ephemeral sources of light such as aurora and fires as well as (non-light) background noise [22]. The radiance for raster fields associated with such ephemeral light or non-light background noise is then set to zero. Hence, we extract the fields of zero radiance for a given annual observation and then set the radiance of these fields to zero for all monthly observations of the following year. This means if the radiance of a raster field in the annual average-masked observation for 2020 is zero, we set the radiance of this field to zero in the monthly observations from January to December 2021. We use preceding annual observations rather than concurrent annual observations to enable the processing of monthly data for 2022 for which no VNL 2.1 annual observation is available at the time of writing. The change to using concurrent annual observations has little impact on the resulting extracted data for 2015–2021.

Following [14], we also introduce an upper bound for the brightness of each raster field at 300 nW ⋅ sr ^−1^ ⋅ cm ^−2^. This threshold reduces outliers associated with disproportionately bright processes, primarily gas flares observed at night. While enforcing this threshold has a negligible effect on the observations in Afghanistan, it is relevant for neighboring countries with larger oil and gas industries, such as Iran.

The monthly cloud-free DNB composite data also includes indicator data which states the number of unobstructed observations for each raster field in a given month. A low number of cloud-free observations is associated with a less reliable observation of the respective raster field for that month. Thus, we introduce a conditional rolling update. For our final processed composite, each raster field is updated with the next month’s radiance value only if the next month includes more than five unobstructed observations of the respective raster field. Otherwise, we keep the radiance value of the previous month.

Given the final, reduced-noise monthly composites, we aggregate radiation by summing over all raster fields in a province. Thus, we construct a time series panel of monthly radiance for Afghanistan, Armenia, Azerbaijan, Bangladesh, Bhutan, Georgia, India, Iran, Iraq, Jordan, Kazakhstan, Kyrgyzstan, Lebanon, Sri Lanka, Nepal, Pakistan, Syria, Tajikistan, Turkmenistan, Turkey, and Uzbekistan. These countries are chosen for their geographic and economic proximity and similarity. Overall, the region of study approximately consists of 5° − 55° N and 25° − 100° E. We consciously excluded some countries from our analysis that fall into this region, yet have very differently structured economies, such as Saudi Arabia, Qatar, and the United Arab Emirates.

**Fig 2:**
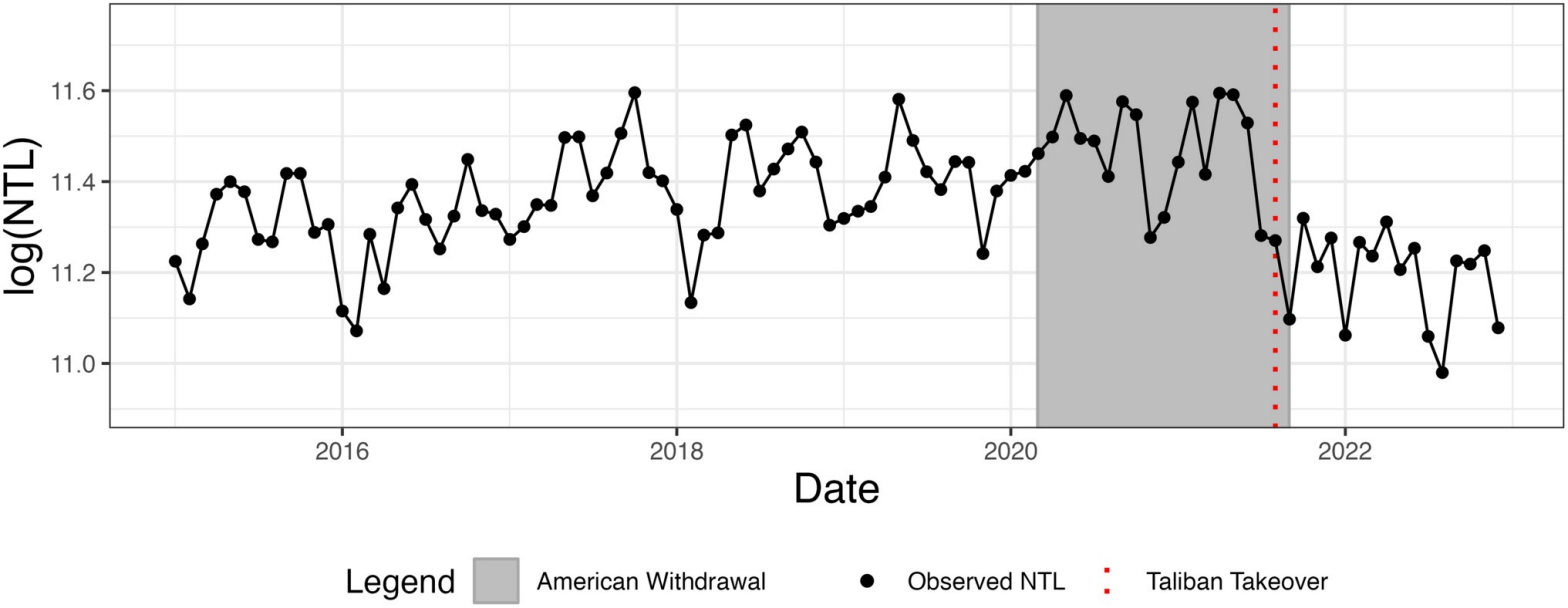
Aggregated extracted nightlight radiance (NTL) for Afghanistan. Aggregated nightlight radiance for Afghanistan based on monthly, reduced-noised observations. The gray period indicates the ongoing American withdrawal from March 2019 to late August 2021 while the red line indicates the Taliban takeover in August 2021.

**Fig 3:**
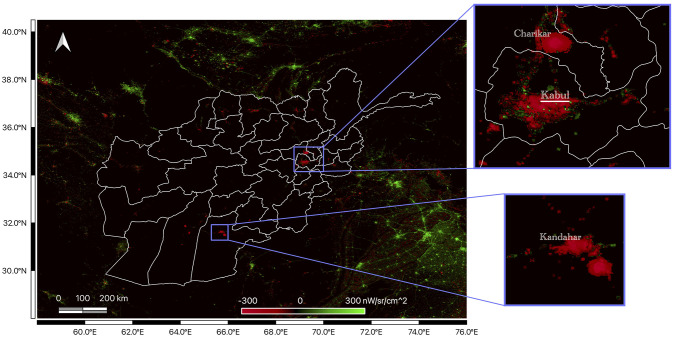
Mapping of the change of nightlight radiance in Afghanistan. Mapping of the difference between the 6-month average radiance from February 2021 to July 2021 and the 6-month average from August 2021 to January 2022. Green and red indicate an increase and decrease in brightness respectively. Inset maps highlight the two most populous regions of Afghanistan: Kabul and Kandahar.

Fig 2 displays the aggregated, logged nightlight radiance for Afghanistan. The dashed vertical line highlights the sudden drop in radiance following the Taliban takeover beginning in mid-2021, which is the change we seek to isolate. Fig 3 shows a map of the change in radiance for Afghanistan between the 6-month average before and after the Taliban takeover. This highlights the decline in radiance in Afghanistan in contrast to neighboring regions. The most populous cities, Kabul and Kandahar, appear to be particularly affected. Note that for different aspects of our empirical analysis, we aggregate Afghanistan’s provincial data at the national level through summation and take non-overlapping averages over quarterly, bi-annual, and annual time frames.

## Methodology

To evaluate the change in Afghanistan’s economy associated with the Taliban takeover in August 2021, we proceed in two steps. First, we construct a counterfactual post-treatment nightlights time series to estimate the impact of the takeover on Afghanistan’s nightlights using the synthetic control approach. Second, we estimate the economic downturn associated with the given change in nightlights using a linear model of the first differences of the log data.

### Synthetic control methodology

We utilize the synthetic control methodology first introduced by [27, 28], and recently expanded by [29] to construct the counterfactual nightlights (NTL) data for Afghanistan. This counterfactual quantifies how Afghanistan’s nightlights might have developed in the absence of the Taliban takeover. Specifically, the synthetic control is a weighted average of provinces of Afghanistan’s neighbors excluding China, chosen to reproduce characteristics of Afghanistan before the takeover. We utilize provinces instead of countries in this weighted average to achieve a more granular and more precise approximation of pre-takeover Afghanistan. Using the terminology of the synthetic control literature, the Taliban takeover is the *treatment*, while *untreated* provinces of Iran, Pakistan, Tajikistan, Turkmenistan, and Uzbekistan constitute the *donor pool*. This prediction interval method allows for using cointegrated time series and, in S4 Table in the Supporting Information, we present evidence of cointegration. Further details on the derivations of the synthetic control prediction and the prediction intervals are included in the S1 Appendix.

### Nowcasting economic output

Next, we introduce the model for estimating the economic downturn associated with the observed reduction of nightlights in Afghanistan. Here, we switch to annual data as for the countries under consideration, GDP is only available on an annual basis. Given the limited data in the intersection of available VIIRS data and GDP data for the countries under consideration, we follow the existing nightlights literature [9, 21] in fiting a parsimonious linear relationship between log GDP and log NTL. However, due to the evidence of non-stationarity reported in S5 Table in the Supporting Information, we are using the first difference, denoted by Δ, of both log GDP and log NTL. Specifically, we utilize the following model

$$\begin{array}{c} \Delta \text{log}\left({\text{GDP}}_{is}\right)=\beta_{0}+\beta_{1}\Delta {\bar{Y}}_{is}+\beta_{2}s+u_{is}\;\;\text{for}\;\;s=1,\ldots ,S\;\text{and}\;i=1,\ldots ,N \end{array}$$ (1)

where *N* is the number of countries under consideration, 1, …, *S* are the years in the range from 2016 to 2022, ${\bar{Y}}_{is}$ denotes the annual average of aggregated log NTL data for country *i* in year *s*, and *u*_*is*_ denotes the error term. Note that we are considering the years from 2016 to 2022 as we lose the first observation due to taking the first difference. Further, using the first difference of the log data approximates percentage changes, effectively removing constant, country-specific differences. Using this model, we nowcast the Δlog(GDP) of Afghanistan for 2021 and 2022. In the results section, we also discuss alternative model specifications, including annual fixed effects, and the performance of random forests and boosted regression trees as benchmarks [30, 31].

## Results

### Synthetic control results

**Fig 4:**
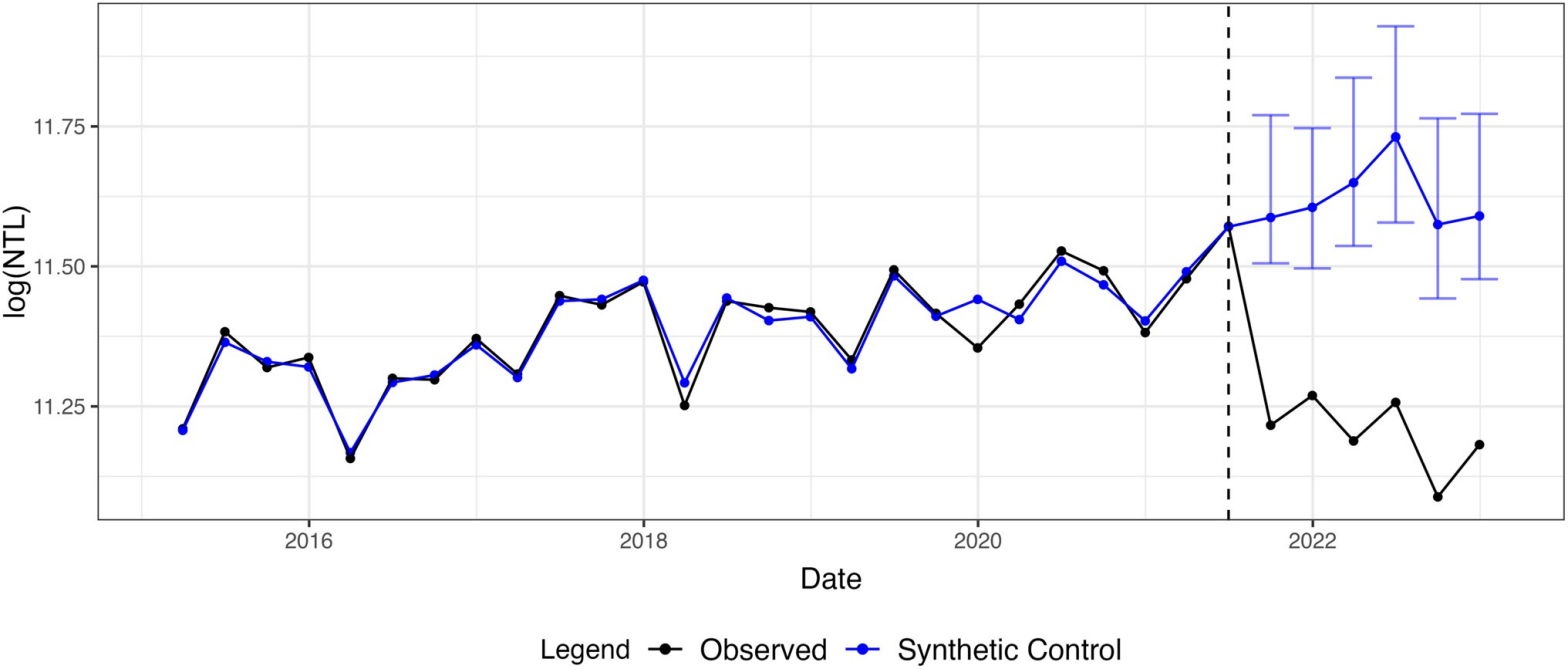
Synthetic control results. Synthetic control (blue) for Afghanistan’s nightlights based on quarterly data (black) from January 2015 to December 2022 including post-treatment prediction intervals for the counterfactual with at least 90% coverage probability.

Fig 4 shows the synthetic control based on quarterly averages of nightlights data to further reduce noise and potential seasonality of the monthly observations. The treatment period is set to be Q2 of 2021, as conservatively defining the treatment period earlier than August 2021 allows for a better fit to the pre-treatment period in the presence of anticipatory effects. The included post-treatment prediction intervals for the counterfactual have at least 90% coverage probability, and the observed drop in Afghanistan’s nightlights consistently lies outside the intervals. We see point estimates of the counterfactual around 11.6 for the log of Afghanistan’s aggregate nightlight radiance, while the observed post-treatment values average approximately 11.2. Thus the average prediction for the treatment effect is approximately 0.4 in terms of log NTL. The Supporting Information section includes corresponding results based on monthly and bi-annual data in S1 Fig and an overview of the non-zero elements of the chosen weights $\hat{w}$ for each model in S1 Table.

### Nowcasting results

Next, we estimate the decline in GDP associated with the reduced nightlights in Afghanistan based on past correlations between GDP and nightlights in the region. We explored three different specifications of linear models, including a time trend and yearly fixed effects, as well as random forest and boosted regression tree benchmark models. S2 Table in the Supporting Information section provides an overview of the results of the three different linear regression models. We used 10-fold cross-validation on the entire dataset and leave-one-out cross-validation, focusing on predicting Afghanistan’s GDP for 2015 to 2020, to assess the out-of-sample prediction accuracy of the different model specifications, as shown in Tables 1 and 2.

**Table 1:** Overview of 10-fold cross-validation performance of the different model specifications.

Model	RMSE	MAE	RMSE SD	MAE SD
(1) NTL	0.14	0.10	0.04	0.02
(2) NTL and time trend	0.13	0.09	0.04	0.02
(3) NTL and yearly fixed effect	0.12	0.09	0.04	0.02
(4) Random Forrest	0.13	0.09	0.04	0.03
(5) Boosted Regression Tree	0.13	0.09	0.04	0.03

All of the reported statistics are the mean values over 10 folds of cross-validation, while SD indicates their standard deviation across those folds.

**Table 2:** Overview of leave one out cross-validation performance, focused on Afghanistan.

Model	RMSE	MAE	RMSE SD	MAE SD
(1) NTL	0.03	0.03	0.01	0.01
(2) NTL and time trend	0.02	0.02	0.01	0.01
(3) NTL and yearly fixed effect	0.06	0.06	0.06	0.06
(4) Random Forrest	0.08	0.08	0.07	0.07
(5) Boosted Regression Tree	0.07	0.07	0.05	0.05

All of the reported statistics are the mean values over the 5 cross-validations while SD indicates their standard deviation across those folds.

While all model specifications achieve comparable performance in the 10-fold cross-validation across all countries, model specifications (1) and (2) perform the best when focusing on predicting Afghanistan’s GDP. This might indicate overfitting of the other models to features that are not as relevant for predicting the GDP changes for Afghanistan. The predictive performance exhibited by model (2) in forecasting Afghanistan’s GDP, coupled with the statistical significance of its explanatory variables, as detailed in S2 Table in the Supporting Information section, motivates our preference for employing this model in the out-of-sample nowcasting of Afghanistan’s GDP for the years 2021 and 2022.

**Fig 5:**
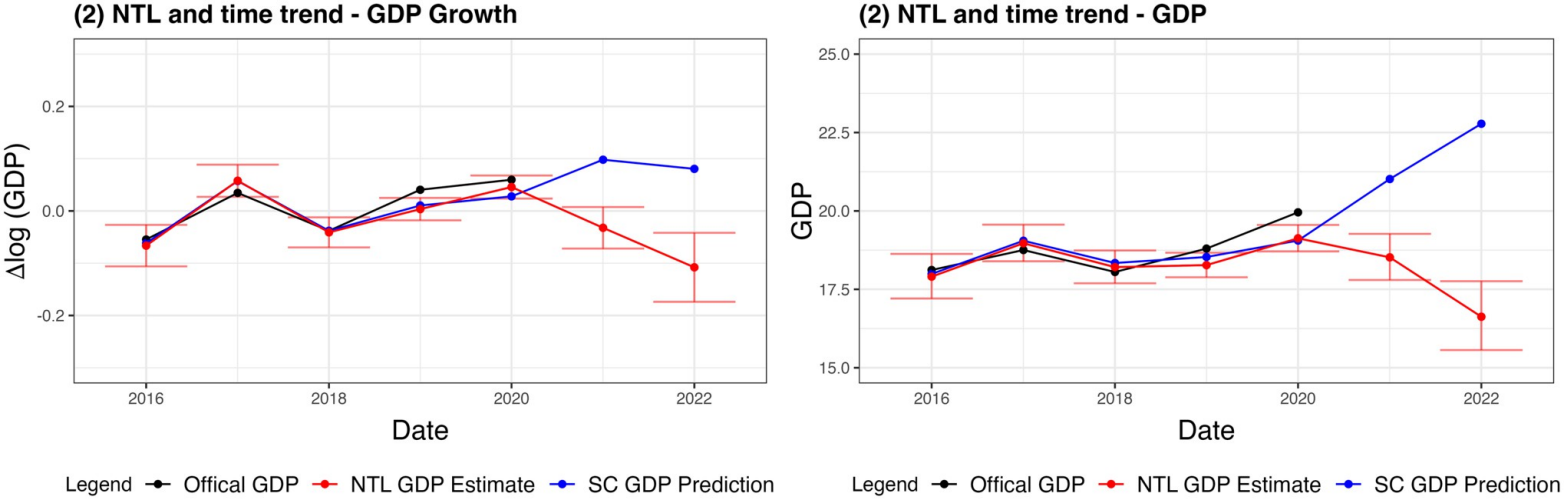
GDP-nowcasting results using model (2). Annual GDP data for Afghanistan as well as estimates based on annual averages of observed NTL radiance including 90% confidence intervals and point estimates based on synthetic control NTL predictions. The figures on the left correspond to the original model specification in terms of Δlog(GDP), while the figure on the right report the same results converted to total GDP USD (billions). The estimates shown are based on model (2) which includes NTL and time trend.

Fig 5 shows the estimated Δlog(GDP) as well as the corresponding total GDP estimates in current USD for Afghanistan based on model (2). For Δlog(GDP), we have predictions of approximately -3% and -10% which corresponds to GDP levels of 18.52 and 16.63 billion USD for 2021 and 2022 respectively. In total, this implies a total decrease of approximately 13% in Afghanistan’s GDP from the inferred 2020 level to the predicted 2022 level and a 16% decrease from the officially reported 2020 level to the predicted 2022 level.

We also report 90% confidence intervals associated with our point estimates. Given the issues of data scarcity and quality, large confidence intervals are to be expected, and indeed this is what we find. Further, the different model specifications lead to notably differing point estimates and confidence intervals as shown in the overview S2 Fig in the Supporting Information section. In particular, model (3) which includes yearly fixed effects appears to predict a strong downturn for 2020, likely due to effects of the Covid epidemic on other countries in the sample that did not manifest in Afghanistan’s official, reported GDP. Still, for our chosen model specification, the confidence intervals for Afghanistan’s GDP changes decisively enter negative territory in 2021 and 2022.

Further, we included the GDP level associated with the annual averages of the point estimates for the synthetic control based on quarterly data. While the exact value of these should be interpreted cautiously due to the accumulated model uncertainty, they serve to highlight the change in the trajectory of Afghanistan’s GDP development. Instead of continuing on the upward trend associated with our counterfactual prediction, this evidence indicates that the economy of Afghanistan has fallen into a deep recession.

## Conclusion

This paper highlights the promise of using monthly nightlights data in estimating regional economic shocks and trends when facing data scarcity or a complete lack of traditional economic indicators. We provide a novel estimate of the economic shock that Afghanistan has suffered by combining nightlights data with the use of the synthetic control methodology. Our methodology stresses the importance of evaluating the change associated with the Taliban take-over relative to the counterfactual growth in the absence of the government’s collapse, rather than simply focusing on the economic level immediately before the takeover.

In this application, we identified a significant fall in the nightlights of Afghanistan and derived an associated point estimate of the downturn in GDP of approximately 16%. This is a notably lower estimate than currently available survey-based measurements. Naturally, this approach comes with its respective limitations, which are primarily driven by data scarcity and noise in both nightlight measurements and GDP reporting in the region. We processed the nightlights to reduce noise and curated a sample of comparable countries in the region to fit our GDP prediction model. However, uncertainty related to the small number of annual pre-treatment observations, in particular for Afghanistan, remains. To quantify some of this uncertainty, we also present confidence intervals and alternative model specifications that suggest caution in interpreting point estimates derived from this setting. Further, the estimated counterfactual growth path might be a slight overestimation due to migration of people and economic activity from Afghanistan to neighboring regions. Still, even with these uncertainties, there is little doubt that Afghanistan has suffered a major economic shock following the Taliban takeover, likely due to reductions in international aid and Western military spending [19]. More specifically, our work serves to isolate the economic shock associated with the Taliban takeover, shifting the country from a positive growth trend to a deep recession.

For many countries beyond Afghanistan, traditional economic data remains unavailable or unreliable, creating a need for innovative approaches to data gathering and analysis. Indeed, with a growing number of countries turning away from democracy, the number of cases of such data-scarce regions may grow [15]. Further, researchers could potentially use this methodology to explore such questions as the effects of Western sanctions on the Russian or Iranian economies. In these settings, the use of relatively high-frequency data such as nightlights enables researchers to get closer to real-time analysis.

When confronted with data scarcity or unreliability, future research might also explore and incorporate additional sources of economic information including call data records of mobile phones [32], social media posts [33], and combinations of such data [12]. These new data sources, alongside the synthetic control methodology, promise more accurate and faster evaluations of regional shocks, providing researchers and policy-makers with a powerful tool in support of economic analysis and potential interventions.

## Supporting information

S1 Fig Overview of synthetic control results. Synthetic control NTL for Afghanistan including post-treatment prediction interval for the counterfactual with at least 90% coverage probability. The treatment period is set to be May 2021 for the model based on monthly data and June of 2021 for the models based on quarterly and bi-annual data.
(TIFF)

S2 Fig Overview of GDP-nowcasting models. Annual GDP data for Afghanistan as well as estimates based on annual averages of observed NTL radiance including 90% confidence intervals and point estimates based on synthetic control NTL predictions. Figures on the left correspond to the original model specification in terms of Δlog(GDP), while figures on the right report the same results converted to total GDP in USD (billions).
(TIFF)

S1 TableEstimates of $\hat{r}$ and nonzero elements of $\hat{\text{w}}$.Intercepts $\hat{r}$ and weighted average vectors $\hat{\text{w}}$ for synthetic controls based on monthly, quarterly, and bi-annual pre-treatment observations (January 2015 to May 2021 / Q1 2015 to Q2 2021) based on model Eq (1).(PDF)

S2 Table Overview of linear GDP models. This overview presents the regression results of the three linear GDP nowcasting models under consideration, relating Δlog(GDP) and Δlog(NTL) from 2016 to 2022.
(PDF)

S3 Table Overview Breusch-Pagan test results for linear GDP models. This overview presents the results of Breusch-Pagan tests on the residuals of the three linear GDP nowcasting models under consideration, relating Δlog(GDP) and Δlog(NTL) from 2016 to 2022. These three tests do not present sufficient evidence to reject the null hypothesis of homoscedasticity [34].
(PDF)

S4 Table Augmented Dickey-Fuller test results for pre-treatment synthetic control residuals. These collected results of different Dickey-Fuller tests run on the pre-treatment residuals of the different synthetic control models present evidence of cointegration of the observed nighttime lights for monthly, quarterly, and bi-annual observations calculated using [35].
(PDF)

S5 Table Augmented Dickey-Fuller test results for GDP and NTL of Afghanistan. These collected results of different Dickey-Fuller tests run on the log(GDP) and log(NTL) time series, as well as their respective first differences, present evidence of unit roots in the original time series and significantly less evidence of unit roots in the first differenced time series. Note that these results are affected by the small sample of annual data ranging from 2015 to 2022. Gaps indicate model specifications with five or fewer remaining time periods, following the default of [35].
(PDF)

S1 Appendix Synthetic control methodology. (PDF)

S1 Data (ZIP)
