# Supplementary figures and images for: Estimating the collapse of Afghanistan’s economy using nightlights data
Source: PLoS One. 2024 Dec 13;19(12):e0315337. doi: 10.1371/journal.pone.0315337 (PMC11642984; doi:10.1371/journal.pone.0315337)

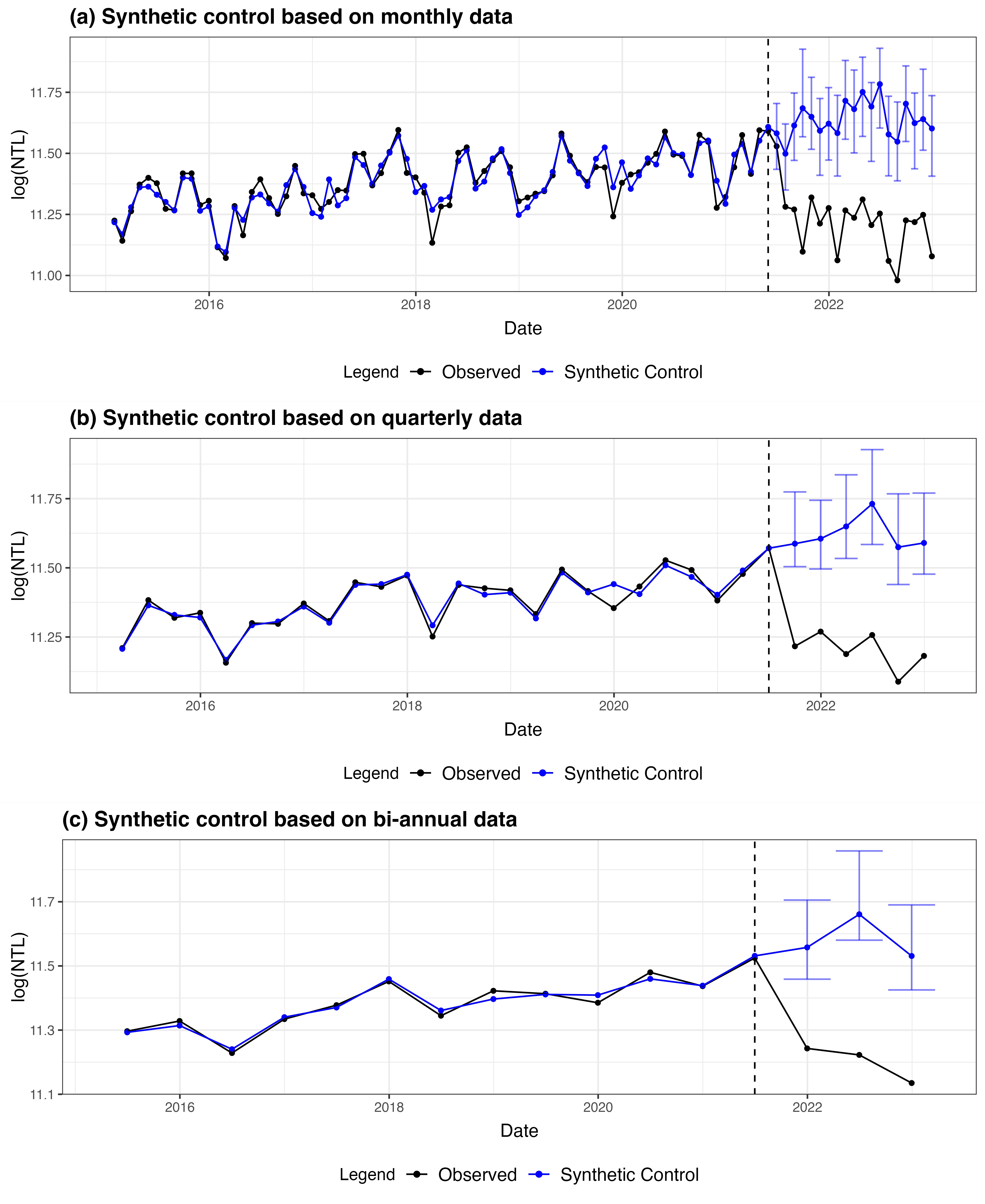

Supplement: S1 Fig — Synthetic control NTL for Afghanistan including post-treatment prediction interval for the counterfactual with at least 90% coverage probability. The treatment period is set to be May 2021 for the model based on monthly data and June of 2021 for the models based on quarterly and bi-annual data. (TIFF) [file pone.0315337.s001.tiff]

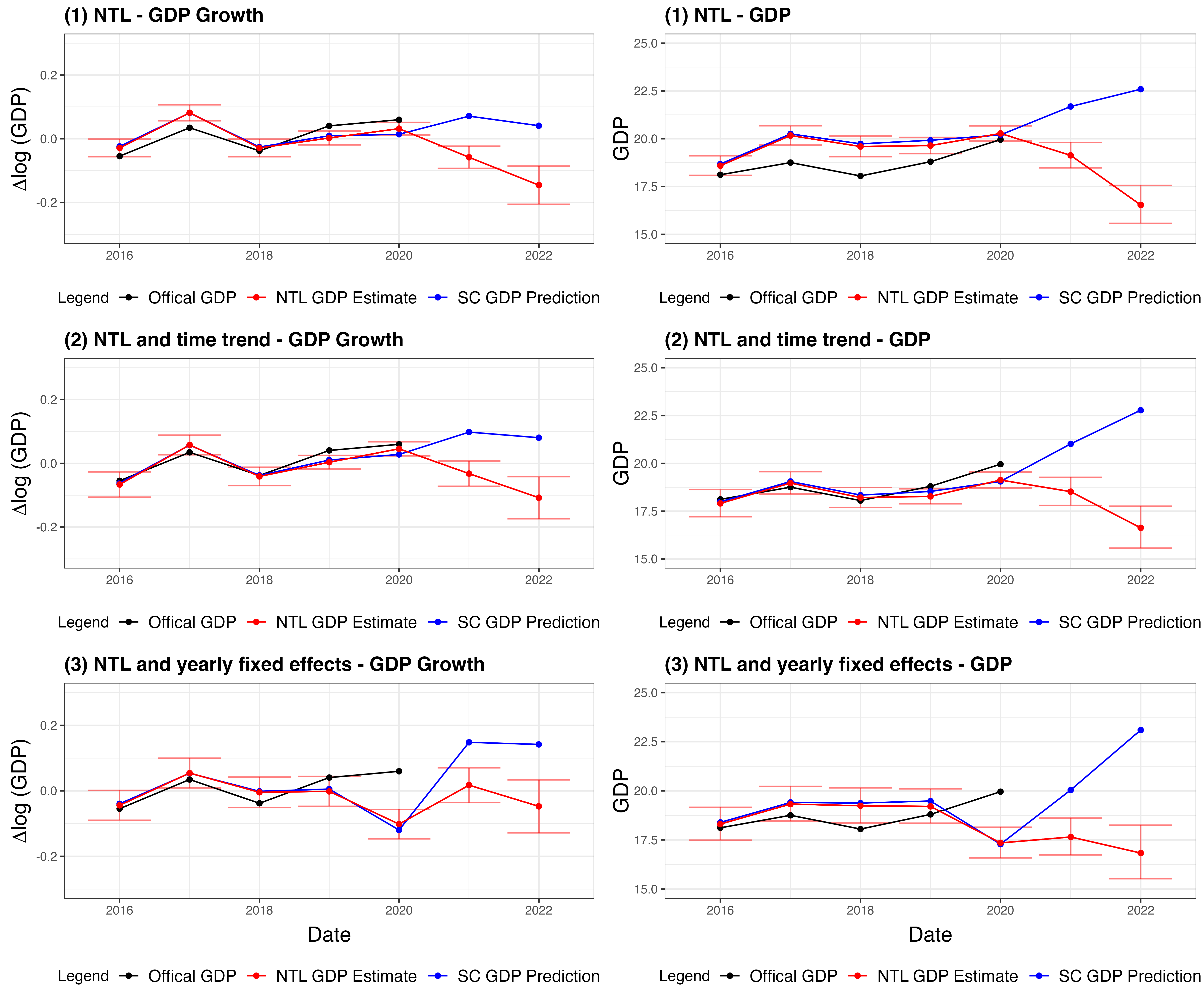

Supplement: S2 Fig — Annual GDP data for Afghanistan as well as estimates based on annual averages of observed NTL radiance including 90% confidence intervals and point estimates based on synthetic control NTL predictions. Figures on the left correspond to the original model specification in terms of Δlog(GDP), while figures on the right report the same results converted to total GDP in USD (billions). (TIFF) [file pone.0315337.s002.tiff]
