# Supplementary material for: Estimating the collapse of Afghanistan’s economy using nightlights data
Source: PLoS One. 2024 Dec 13;19(12):e0315337. doi: 10.1371/journal.pone.0315337 (PMC11642984; doi:10.1371/journal.pone.0315337)
Supplement: S1 Table — Intercepts r^ and weighted average vectors w^ for synthetic controls based on monthly, quarterly, and bi-annual pre-treatment observations (January 2015 to May 2021 / Q1 2015 to Q2 2021) based on model Eq (1). (PDF) [file pone.0315337.s003.pdf]

Table 1. Estimates of $\widehat{r}$ and nonzero elements of $\widehat{\mathbf{w}}$

Country	Province	Monthly data	Quarterly data	Bi-annual data
Intercept	-	1.29	1.50	0.96
Iran	Hormozgan	0.06	0.00	0.00
Iran	Tehran	0.02	0.00	0.00
Iran	West Azarbaijan	0.00	0.00	0.11
Iran	Bushehr	0.03	0.01	0.00
Iran	Zanjan	0.00	0.02	0.00
Pakistan	F.A.T.A.	0.17	0.20	0.19
Pakistan	Northern Areas	0.09	0.08	0.01
Tajikistan	Dushanbe	0.25	0.22	0.05
Tajikistan	Khatlon	0.01	0.00	0.04
Tajikistan	Tadzhikistan Territories	0.00	0.01	0.00
Turkmenistan	Ashgabat	0.09	0.18	0.17
Turkmenistan	Chardzhou	0.05	0.00	0.00
Turkmenistan	Mary	0.04	0.00	0.00
Uzbekistan	Andijon	0.05	0.02	0.11
Uzbekistan	Sirdaryo	0.00	0.00	0.01
Uzbekistan	Tashkent City	0.00	0.00	0.12
Uzbekistan	Bukhoro	0.05	0.00	0.00
Uzbekistan	Kashkadarya	0.00	0.07	0.11
Uzbekistan	Namangan	0.08	0.18	0.09
