# Supplementary material for: Estimating the collapse of Afghanistan’s economy using nightlights data
Source: PLoS One. 2024 Dec 13;19(12):e0315337. doi: 10.1371/journal.pone.0315337 (PMC11642984; doi:10.1371/journal.pone.0315337)
Supplement: S2 Table — This overview presents the regression results of the three linear GDP nowcasting models under consideration, relating Δlog(GDP) and Δlog(NTL) from 2016 to 2022. (PDF) [file pone.0315337.s004.pdf]

Table 2. Overview of linear GDP models

	(1) NTL	(2) NTL and time trend	(3) NTL and yearly fixed effect
(Intercept)	-0.01 (0.01)	-0.05 (0.02)*	-0.02 (0.03)
$\Delta \log$(NTL)	0.67 (0.13)***	0.68 (0.13)***	0.68 (0.14)***
year		0.01 (0.01)*	
factor(2017)			-0.01 (0.04)
factor(2018)			0.04 (0.04)
factor(2019)			0.01 (0.04)
factor(2020)			-0.12 (0.04)**
factor(2021)			0.09 (0.04)*
factor(2022)			0.12 (0.04)**
R^2	0.16	0.19	0.37
Adj. R^2	0.16	0.18	0.34
Num. obs.	142	142	142

$^{***}p < 0.001$; $^{**}p < 0.01$; $^{*}p < 0.05$
