# Supplementary material for: Estimating the collapse of Afghanistan’s economy using nightlights data
Source: PLoS One. 2024 Dec 13;19(12):e0315337. doi: 10.1371/journal.pone.0315337 (PMC11642984; doi:10.1371/journal.pone.0315337)
Supplement: S3 Table — This overview presents the results of Breusch-Pagan tests on the residuals of the three linear GDP nowcasting models under consideration, relating Δlog(GDP) and Δlog(NTL) from 2016 to 2022. These three tests do not present sufficient evidence to reject the null hypothesis of homoscedasticity [34]. (PDF) [file pone.0315337.s005.pdf]

Table 3. Overview Breusch-Pagan test results for linear GDP models

	(1) NTL	(2) NTL and time trend	(3) NTL and yearly fixed effect
Test Statistic	2.01	2.61	7.29
Degrees of freedom	1	2	7
p-value	0.16	0.27	0.40
