# Supplementary material for: Estimating the collapse of Afghanistan’s economy using nightlights data
Source: PLoS One. 2024 Dec 13;19(12):e0315337. doi: 10.1371/journal.pone.0315337 (PMC11642984; doi:10.1371/journal.pone.0315337)
Supplement: S4 Table — These collected results of different Dickey-Fuller tests run on the pre-treatment residuals of the different synthetic control models present evidence of cointegration of the observed nighttime lights for monthly, quarterly, and bi-annual observations calculated using [35]. (PDF) [file pone.0315337.s006.pdf]

Table 4. Augmented Dickey-Fuller test results for pre-treatment synthetic control residuals

(a) Type 1: No drift, no trend

	Monthly		Quarterly		Bi-annual	
lag	ADF	p.value	ADF	p.value	ADF	p.value
0	-7.83	0.01	-5.68	0.01	-4.99	0.01
1	-6.09	0.01	-4.21	0.01	-8.17	0.01
2	-4.99	0.01	-4.81	0.01	-2.17	0.03

(b) Type 2: Including drift, no trend

	Monthly		Quarterly		Bi-annual	
lag	ADF	p.value	ADF	p.value	ADF	p.value
0	-7.78	0.01	-5.56	0.01	-4.75	0.01
1	-6.04	0.01	-4.11	0.01	-7.72	0.01
2	-4.95	0.01	-4.68	0.01	-2.05	0.31

(c) Type 3: Including drift and trend

	Monthly		Quarterly		Bi-annual	
lag	ADF	p.value	ADF	p.value	ADF	p.value
0	-7.80	0.01	-5.50	0.01	-4.51	0.01
1	-6.13	0.01	-4.03	0.02	-7.79	0.01
2	-5.08	0.01	-4.61	0.01	-2.30	0.44
