# Supplementary material for: Estimating the collapse of Afghanistan’s economy using nightlights data
Source: PLoS One. 2024 Dec 13;19(12):e0315337. doi: 10.1371/journal.pone.0315337 (PMC11642984; doi:10.1371/journal.pone.0315337)
Supplement: S5 Table — These collected results of different Dickey-Fuller tests run on the log(GDP) and log(NTL) time series, as well as their respective first differences, present evidence of unit roots in the original time series and significantly less evidence of unit roots in the first differenced time series. Note that these results are affected by the small sample of annual data ranging from 2015 to 2022. Gaps indicate model specifications with five or fewer remaining time periods, following the default of [35]. (PDF) [file pone.0315337.s007.pdf]

Table 5. Augmented Dickey-Fuller test results for GDP and NTL of Afghanistan

(a) Type 1: No drift, no trend

	log(GDP)		log(NTL)		Δ log(GDP)		Δ log(NTL)	
lag	ADF	p.value	ADF	p.value	ADF	p.value	ADF	p.value
0	-0.20	0.58	-0.92	0.34	-1.87	0.06	-1.78	0.07
1	0.43	0.76	-0.41	0.52	-1.56	0.11	-1.95	0.05
2	0.70	0.84	-0.33	0.54			-0.04	0.63

(b) Type 2: Including drift, no trend

	log(GDP)		log(NTL)		Δ log(GDP)		Δ log(NTL)	
lag	ADF	p.value	ADF	p.value	ADF	p.value	ADF	p.value
0	-2.26	0.24	-1.70	0.44	-1.41	0.54	-1.69	0.44
1	-1.44	0.53	-2.07	0.31	-0.72	0.78	-1.66	0.45
2			-0.90	0.72			1.54	0.99

(c) Type 3: Including drift and trend

	log(GDP)		log(NTL)		Δ log(GDP)		Δ log(NTL)	
lag	ADF	p.value	ADF	p.value	ADF	p.value	ADF	p.value
0	-0.95	0.93	-1.46	0.77	-3.21	0.11	-1.53	0.74
1	-0.52	0.97	-0.87	0.94			-1.64	0.70
2			2.00	0.99				
