# Supplementary material for: Estimating the collapse of Afghanistan’s economy using nightlights data
Source: PLoS One. 2024 Dec 13;19(12):e0315337. doi: 10.1371/journal.pone.0315337 (PMC11642984; doi:10.1371/journal.pone.0315337)
Supplement: S1 Appendix — (PDF) [file pone.0315337.s008.pdf]

Appendix

Synthetic Control Methodology

Following the common notation and the setup in [1], let $i \in \{1, \ldots J+1\}$ be the regions whose aggregated log NTL, denoted Y_{it}, we observed. Here, $i = 1$ denotes Afghanistan, and thus $i \in \{2, ..., J+1\}$ corresponds to the J untreated regions. Denote the time periods for which we observed Y_{it} by $t \in \{1, \ldots, T\}$ and let $t = T_0$ be the time period of Taliban takeover. For each region i and time period t, let $Y_{it}(0)$ and $Y_{it}(1)$ denote the outcome in the absence of treatment and under treatment respectively. Thus, the observed outcomes are given by

$$Y_{it} = \begin{cases} Y_{it}(0), & \text{if} \quad i \in \{2, \ldots, J+1\} \text{ and } t \in \{1, \ldots, T\} \\ Y_{it}(0), & \text{if} \quad i = 1 \text{ and } t \in \{1, \ldots, T_0\} \\ Y_{it}(1), & \text{if} \quad i = 1 \text{ and } t \in \{T_0 + 1, \ldots, T\}. \end{cases}$$

This allows us to express the treatment effect of the Taliban takeover on Afghanistan's NTL as follows:

$$\tau_t := Y_{1t}(1) - Y_{1t}(0), \quad t > T_0. \tag{1}$$

Step 1 - Prediction:

To extract τ_t from the expression above, it is imperative to construct a good prediction of the counterfactual $Y_{1t}(0)$ for $t \in \{T_0 + 1, \ldots, T\}$ which corresponds to NTL in Afghanistan if the takeover had not taken place.

To construct this prediction, let $\mathbf{y} \in \mathbb{R}^{T_0}$ denote the pre-treatment NTL observations of Afghanistan and let $\mathbf{X} \in \mathbb{R}^{T_0 \times J}$ denote the pre-treatment NTL observations of the donor pool with $\mathbf{X}_t$ denoting its t-th row. This allows us to fit

$$\begin{pmatrix} \widehat{\mathbf{w}} \\ \widehat{r} \end{pmatrix} \in \underset{\mathbf{w} \in \mathcal{W}, \mathbf{r} \in \mathbb{R}}{\arg\min} ||\mathbf{y} - \mathbf{X}\mathbf{w} - r||_2^2, \tag{2}$$

where $\mathcal{W} := \{\mathbf{w} \in \mathbb{R}_+^J : \|\mathbf{w}\|_1 = 1\}$. Thus $\widehat{\mathbf{w}}$ are the weights used to construct the weighted average and $\widehat{r}$ is an intercept parameter essentially corresponding to a fixed effect for Afghanistan. The weights vector $\widehat{\mathbf{w}}$ is constrained to be within $\mathcal{W}$ to achieve sparsity, as well as to prevent overfitting and implausible relationships such as $\mathrm{w}_i < 0$. Following the average-weighted model Eq (2), the out-of-sample predictions for the counterfactual are given by

$$\widehat{Y}_{1t}(0) = \widehat{r} + \sum_{i=2}^{J+1} \widehat{\mathrm{w}}_i Y_{it}(0) = \widehat{r} + \mathbf{X}_t \cdot \widehat{\mathbf{w}} \quad \text{for} \quad t \in \{T_0 + 1, \ldots, T\}.$$

Step 2 - Prediction Intervals:

To validate empirically that the predicted treatment effect $\widehat{\tau}_t$ based on Eq (1) is statistically significant, we adopt the recently proposed methodology of [2] and software of [3] to construct prediction intervals.

If $\widehat{\mathbf{w}}$ and $\widehat{r}$ are concentrating around $\mathbf{w}_0 \in \mathcal{W}$ and $r_0 \in \mathbb{R}$ respectively, we can express the total uncertainty regarding the prediction of the treatment effect in two parts:

$$\widehat{\tau}_t - \tau_t = Y_{1t}(0) - \widehat{Y}_{1t}(0) = e_t - ((\widehat{r} - r_0) + \mathbf{X}_t \cdot (\widehat{\mathbf{w}} - \mathbf{w}_0)) \quad \text{for} \quad t \in \{T_0 + 1, \ldots, T\}.$$

where $e_t := Y_{1t}(0) - r_0 - \mathbf{X}_t \cdot \mathbf{w}_0$. The first part is the out-of-sample error e_t related to potential misspecification along with any additional noise occurring during the post-treatment periods $t > T_0$. For our implementation, we assumed a low probability for large out-of-sample prediction errors, and thus assumed e_t to be sub-Gaussian.

The second part captures the in-sample uncertainty $((\widehat{r} - r_0) + \mathbf{X}_t \cdot (\widehat{\mathbf{w}} - \mathbf{w}_0))$ stemming from the construction of $\widehat{\mathbf{w}}$ and $\widehat{r}$ in Eq (2) based on pre-treatment data which is carried over into the prediction $Y_{1t}(0)$ for $t > T_0$. This uncertainty is quantified by estimating the covariance matrix of the in-sample residuals

$$\epsilon_t := Y_{1t}(0) - \widehat{Y}_{1t}(0) = Y_{1t}(0) - \widehat{r} - \mathbf{X}_t \cdot \widehat{\mathbf{w}} \quad \text{for} \quad t \in \{1, \ldots, T_0\},$$

re-sampling this error term 500 times, and estimating quantiles for the associated difference between $\widehat{\mathbf{w}},\ \widehat{r}$ and $\mathbf{w}_0,\ r_0$. Estimating these two sources of uncertainty separately and combining them using the union bound allows for the construction of the prediction interval for τ_t. We refer to [1] for a detailed derivation of the prediction intervals.

References

1. Cattaneo MD, Feng Y, Titiunik R. Prediction intervals for synthetic control methods. Journal of the American Statistical Association. 2021;116(536):1865-80.

2. Cattaneo MD, Feng Y, Palomba F, Titiunik R. `scpi`: Uncertainty Quantification for Synthetic Control Estimators. arXiv preprint arXiv:220205984. 2022.

3. Cattaneo M, Feng Y, Palomba F, Titiunik R. `scpi`: Prediction Intervals for Synthetic Control Methods with Multiple Treated Units and Staggered Adoption; 2022. R package version 2.0.0. `https://CRAN.R-project.org/package=scpi`.
